# T-cell reconstitution during murine acquired immunodeficiency syndrome (MAIDS) produces neuroinflammation and mortality in animals harboring opportunistic viral brain infection

**DOI:** 10.1186/1742-2094-10-98

**Published:** 2013-07-31

**Authors:** Manohar B Mutnal, Scott J Schachtele, Shuxian Hu, James R Lokensgard

**Affiliations:** 1 Neuroimmunology Laboratory, Center for Infectious Diseases and Microbiology Translational Research, Department of Medicine, University of Minnesota, 3-220 LRB/MTRF, 2001 6th Street S.E., Minneapolis, MN 55455, USA

## Abstract

**Background:**

Highly active antiretroviral therapy (HAART) restores inflammatory immune responses in AIDS patients which may unmask previous subclinical infections or paradoxically exacerbate symptoms of opportunistic infections. In resource-poor settings, 25% of patients receiving HAART may develop CNS-related immune reconstitution inflammatory syndrome (IRIS). Here we describe a reliable mouse model to study underlying immunopathological mechanisms of CNS-IRIS.

**Methods:**

Utilizing our HSV brain infection model and mice with MAIDS, we investigated the effect of immune reconstitution on MAIDS mice harboring opportunistic viral brain infection. Using multi-color flow cytometry, we quantitatively measured the cellular infiltrate and microglial activation.

**Results:**

Infection with the LP-BM5 retroviral mixture was found to confer susceptibility to herpes simplex virus (HSV)-1 brain infection to normally-resistant C57BL/6 mice. Increased susceptibility to brain infection was due to severe immunodeficiency at 8 wks p.i. and a marked increase in programmed death-1 (PD-1) expression on CD4^+^ and CD8^+^ T-cells. Both T-cell loss and opportunistic brain infection were associated with high level PD-1 expression because PD-1-knockout mice infected with LP-BM5 did not exhibit lymphopenia and retained resistance to HSV-1. In addition, HSV-infection of MAIDS mice stimulated peripheral immune cell infiltration into the brain and its ensuing microglial activation. Interestingly, while opportunistic herpes virus brain infection of C57BL/6 MAIDS mice was not itself lethal, when T-cell immunity was reconstituted through adoptive transfer of virus-specific CD3^+^ T-cells, it resulted in significant mortality among recipients. This immune reconstitution-induced mortality was associated with exacerbated neuroinflammation, as determined by MHC class II expression on resident microglia and elevated levels of Th1 cytokines in the brain.

**Conclusions:**

Taken together, these results indicate development of an immune reconstitution disease within the central nervous system (CNS-IRD). Experimental immune reconstitution disease of the CNS using T-cell repopulation of lymphopenic murine hosts harboring opportunistic brain infections may help elucidate neuroimmunoregulatory networks that produce CNS-IRIS in patients initiating HAART.

## Background

Highly active antiretroviral therapy (HAART) has transformed HIV-induced disease from a fatal infection to a chronic yet manageable condition. HAART has led to dramatic reductions in plasma viral load, improvement in CD4^+^ T-cell counts, and partial restoration of overall immune function. These immunological changes correlate with reduced frequency of opportunistic infections (OI) and prolonged survival [1,2]. However, a subgroup of patients experience paradoxical clinical deterioration as a consequence of rapid, dysregulated restoration of antigen-specific immune responses following the initiation of antiretroviral treatment [3]. This was first noted following introduction of zidovudine monotherapy in the early 1990s, when localized forms of *Mycobacterium avium-intracellulare* infection were observed in association with recovery rather than failure of cellular immune responses [4]. Over the past two decades, symptomatic deterioration in patients initiating HAART has been described in relation to a number of pre-existing subclinical infections, inflammatory disorders, and autoimmune diseases. This phenomenon is known by a multitude of names including, immune reconstitution inflammatory syndrome (IRIS), immune reconstitution or restoration disease (IRD), and immune reconstitution syndrome (IRS), however, all these names refer to a disease process produced by the reemergence of functional immune responses, as opposed to disorders due to immune suppression [5].

IRIS occurs in approximately 20 to 35% of HIV patients treated with HAART [6], of which an estimated 1% develop central nervous system (CNS)-related IRIS [7]. Up to 28% of patients starting therapy in resource-limited settings may develop CNS-IRIS [8]. The immunopathogenesis of CNS-IRIS is unclear and there is a paucity of literature due to lack of available animal models. Although a wide variety of opportunistic pathogens have been associated with CNS-IRIS, common immunopathological mechanisms are suspected of driving the disproportionate neuroinflammation defining this disease. Proposed mechanisms of CNS-IRIS suggest that dysregulated neuroimmune responses to a variety of antigenic stimuli following initiation of therapy produce disease. The antigenic stimulus in infectious conditions may be either intact viable organisms or dead organisms along with their residual antigens, whereas autoimmune responses to innate antigens are involved in non-infectious causes. The pathophysiology is believed to involve a combination of factors, including reconstitution of immune cell numbers and function, redistribution of lymphocytes, defects in regulatory function, changes in T-helper (Th) cell profiles, underlying antigenic burden, and host genetic susceptibility [9].

Diagnosis of CNS-IRIS is challenging due to varying severity of clinical presentation as well as limited access to the CNS. The ideal criteria for diagnosing CNS-IRIS include a history of HIV infection, worsening of clinical neurological status, either new neuroradiological findings or deterioration of previous findings unexplainable by previous illness or therapy, a log-fold or greater decrease in viral load, and HAART often with increasing CD4^+^ cell counts. If available, histopathologic findings demonstrating T-cell infiltrates into the CNS confirm a CNS-IRIS diagnosis [10].

Because humans are the only natural hosts for HIV, a limited number of approaches are available to study CNS-IRIS. Susceptible strains of mice inoculated as adults with the LP-BM5 murine leukemia virus (MuLV) mixture develop a syndrome termed murine acquired immunodeficiency syndrome (MAIDS). Although not completely analogous to AIDS, many features of MAIDS resemble those observed in individuals infected with HIV, including polyclonal B-cell activation, hypergammaglobulinemia, enhanced susceptibility to infection, profoundly decreased T- and B-cell responses, increased susceptibility to opportunistic pathogens, and the development of terminal B-cell lymphomas [11,12]. Despite definite differences between LP-BM5 and HIV-1, decades of research have established that LP-BM5 infection in susceptible C57BL/6 mice induces an HIV-like immunodeficiency syndrome, hence the term murine AIDS.

Immunopathogenic mechanisms resulting in CNS-IRIS are difficult to address through clinical studies. In the current study, we explored the possibility of developing a small-animal model to address these mechanisms. We found that C57BL/6 mice infected with LP-BM5 displayed profound lymphopenia and exhausted immune responses as measured by programmed death-1 (PD-1) expression on T-cells, and became sensitive to opportunistic herpes simplex virus (HSV)-1 brain infection. Opportunistic viral infection of the brain was associated with T-cell exhaustion, as PD-1 knockout (KO) mice infected with LP-BM5 retained resistance to opportunistic infection. Further, T-cell reconstitution was found to be lethal in MAIDS mice harboring OI of the brain. This mortality was associated with increased microglial activation and elevated levels of proinflammatory cytokine expression within the brain. This model will be useful in further elucidating the mechanisms responsible for HIV-associated CNS-IRIS.

## Methods

### Ethical approval

This study was carried out in strict accordance with recommendations in the Guide for the Care and Use of Laboratory Animals of the National Institutes of Health. The protocol was approved by the Institutional Animal Care and Use Committee (Protocol Number: 1105A99494) of the University of Minnesota.

### Viruses and animals

The LP-BM5 retrovirus mixture was procured from the NIH AIDS reagent program (Germantown, MD, USA). LP-BM5 viral stocks were prepared as described previously [13]. Virus stocks used for infection were produced as cell-free supernatants of SC-1 cells. Titers were determined by a standard retroviral XC plaque assay for the BM5eco virus. C57BL/6 (Charles River Laboratories, Wilmington, MA, USA) or PD-1 KO (provided by Sing-Sing Way, University of Minnesota, Minneapolis, MN, USA) female mice were inoculated via the intraperitoneal (i.p.) route with two doses (2 × 10^4^/PFU dose) in 250 μl, with 3 d between doses [13].

### HSV-1

HSV-1 strain 17syn^+^, a neurovirulent strain of HSV-1, provided by LT Feldman (University of California, Los Angeles, CA, USA) was used in all experiments. The virus was propagated in rabbit skin fibroblasts (CCL68; American Type Culture Collection), sucrose purified, and titered using standard plaque assay. MAIDS mice were challenged intranasally (i.n.) with 2.0 × 10^5^ PFU/mouse at various time points to examine their susceptibility to HSV brain infection.

### Monitoring of LP-BM5 infection

Mice infected with LP-BM5 were monitored by physical examination for cervical lymphadenopathy and by bleeding at various time points. Blood collected through facial vein puncture was lysed using RBS lysis buffer (ACK lysing buffer, Life Technologies, Grand Island, NY, USA). Peripheral blood mononuclear cells (PBMC) thus prepared were stained with Mabs CD4-eflour450, CD8-PE-Cy7, PD-1-FITC, CD3-APC-Cy7 (ebioscience, San Diego, CA, USA) and analyzed using flow cytometry.

### Isolation of brain leukocytes and fluorescence-activated cell sorting (FACS)

Leukocytes were isolated from different groups of mice infected with either LP-BM5 or HSV, or dual infection using a previously described procedure with minor modifications [14,15]. In brief, brain tissues harvested from four to six animals were minced finely in Roswell Park Memorial Institute (RPMI) medium 1640 (2 g/L D-glucose and 10 mM 4-(2-hydroxyethyl)-1-piperazineethanesulfonic acid (HEPES)) and digested in 0.0625% trypsin (in Ca/Mg-free Hank’s balanced salt solution (HBSS)) at room temperature for 20 minutes. Single cell preparations from infected brains were resuspended in 30% Percoll and banded on a 70% Percoll cushion at 900 × g at 15°C. Brain leukocytes obtained from the 30 to 70% Percoll interface were treated with Fc Block (anti-CD32/CD16 in the form of 2.4G2 hybridoma culture supernatant with 2% normal rat and 2% normal mouse serum) to inhibit nonspecific antibody (Ab) binding and were stained with anti-mouse immune cell surface markers for 45 minutes at 4°C (anti-CD45-PE-Cy5, anti-CD11b-AF700, anti-CD4-eflour450, anti-major histocompatability complex (MHC) class II-allophycocyanin (APC), anti-CD8-PE-Cy7, and anti-CD3-APC-Cy7 (ebioscience, San Diego, CA, USA) and analyzed by flow cytometry. Control isotype Abs were used for all isotype and fluorochrome combinations to assess nonspecific Ab binding. Live leukocytes were gated using forward scatter and side scatter parameters on a BD FACSCanto flow cytometer (BD Biosciences, San Jose, CA, USA). Data were analyzed using FlowJo software (TreeStar, Ashland, OR, USA).

### Adoptive transfer

Spleen and lymph nodes (cervical, lumbar, mesenteric and inguinal) from HSV-primed (1 × 10^4^ PFU/mouse, i.p. injection) donor animals were collected aseptically at 7 d post-priming. Single cell suspensions of immunocytes were depleted of red blood cells (RBC) by treatment with 0.87% ammonium chloride and washed twice, and cell viability was confirmed using trypan blue. CD3^+^ lymphocytes were enriched by negative selection using a CD3^+^ cell purification kit, as per the manufacturer’s instructions (R&D systems, Minneapolis, MN USA). Immune cells were transferred (2 × 10^6^ or 5 × 10^6^ cells/mouse) into MAIDS mice via the tail vein 7 d post-infection (p.i.) with HSV.

### Real-time PCR

Total RNA was extracted from brain tissue homogenates using the Trizol reagent (Invitrogen, Carlsbad, CA, USA). One μg RNA was DNase (Ambion, Applied Biosystems, Austin, TX, USA) treated, and reverse transcribed to cDNA with SuperScript™ III (Invitrogen), dNTP (GE Healthcare, Piscataway, NJ, USA) and oligo (dT)_12–18_ (Promega, Madison, WI, USA). Real-time PCR was performed in an Mx3000p (Stratagene, La Jolla, CA, USA) with SYBR Advantage qPCR Premix (Clontech, Mountain View, CA, USA), primers and cDNA according to the manufacturer’s protocol. Reaction conditions for quantitative PCR (qPCR) were as follows: initial denaturation at 95°C for 15 sec, amplification for 40 cycles at 95°C for 10 sec, 60°C for 10 sec and 72°C for 10 sec followed by dissociation curve analysis (one cycle at 95°C for 60 sec, 55°C for 30 sec and 95°C for 30 sec) to verify product specificity. After normalizing to hypoxanthine guanine phosphoribosyl transferase-1 (HPRT-1) expression (Δ cycle threshold (Ct) = target gene Ct - HPRT Ct) and then to the control group (ΔΔCt = treatment ΔCt - C ΔCt), relative quantification using 2^∧−ΔΔCt^ was calculated as fold change of target mRNA expression versus control. Primer sequences for HPRT, HSV glyD, IL-2, IFN-γ, inducible nitric oxide synthase (iNOS), chemokine (C-X-C motif) ligand (CXCL)9 and CXCL10 will be available upon request. For HSV glyD expression, samples with Ct values below 35 cycles were identified as positive. Expression was validated by running all samples on a 2% agarose electrophoresis to confirm presence of the PCR product (208 bp).

### Virus recovery

Brains isolated from HSV-infected MAIDS mice at 7 d p.i. were divided into cortex, sub-cortex, cerebellum, and brain stem. Tissue samples were homogenized in DMEM with 5% FBS and stored at −80°C until they were processed. Fifty μL of tissue homogenate was then inoculated onto previously prepared rabbit skin fibroblast cultures in a 24-well plate. The cultures were maintained in DMEM supplemented with 10% FBS and antibiotics. The cultures were analyzed microscopically for the occurrence of cytopathic effect (CPE) and maintained for a period of 7 d before reporting.

## Results

### Lymphopenia during chronic murine retroviral infection

**Figure 1:**
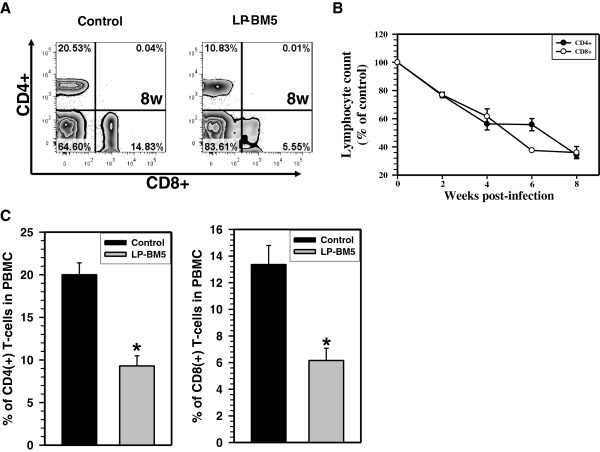
**Development of lymphopenia during murine acquired immunodeficiency syndrome.** C57BL/6 mice were infected with the LP-BM5 retrovirus mixture and disease progression was assessed by determining the percentage of lymphocytes in peripheral blood at the indicated time points. Peripheral blood mononuclear cells (PBMC) were isolated and stained with the mAbs CD3-APC-Cy7, CD4-eFlour450, and CD8-PE-Cy7 for flow cytometry. **(A)** Representative flow cytometry contour plots of data from peripheral blood mononuclear cells show the ratio of CD4^+^ to CD8^+^ T-cells at 8 w post-infection (p.i.). **(B)** The numbers of CD4^+^ and CD8^+^ T-cells in peripheral blood samples obtained from infected animals were determined at the indicated time points and expressed as the percent of uninfected control animals. (**C**) The percentage of CD4^+^ and CD8^+^ T-cells in PBMC at the 8 w p.i. time point are shown. Data are presented as mean (± standard error) percent of control T-cells pooled from three independent experiments. ^*^P <0.01 versus uninfected littermates.

We first examined numbers of T-lymphocytes in peripheral blood at various time points following LP-BM5 infection. Representative flow cytometry plots in Figure 1A show that LP-BM5 infection of C57BL/6 mice produced a sharp decline in numbers of circulating CD4^+^ and CD8^+^ T-cells by 8 w p.i. LP-BM5-infected mice showed an approximately 50% decline in CD4^+^ T-cells when compared to uninfected littermates. A striking fall in CD8^+^ T-cell numbers was also observed at 8 w p.i., demonstrating that these animals were lymphopenic. LP-BM5-infected mice were then monitored from 2 to 8 w p.i. and data presented in Figure 1B indicate a gradual loss of CD4^+^ and CD8^+^ T-cells that reached its maximum (>60%) decline by 8 w p.i. Data shown in Figure 1C indicate the percentage of CD4^+^ (20% ± 1.14 versus 9.3% ± 1.19) and CD8^+^ (13.3% ± 1.44 versus 6.1% ± 0.9) T-cells from three pooled independent experiments and display a significant decrease in the number of cells at 8 w p.i., when compared to uninfected littermates.

### Elevated levels of PD-1 on leukocytes during chronic infection

**Figure 2:**
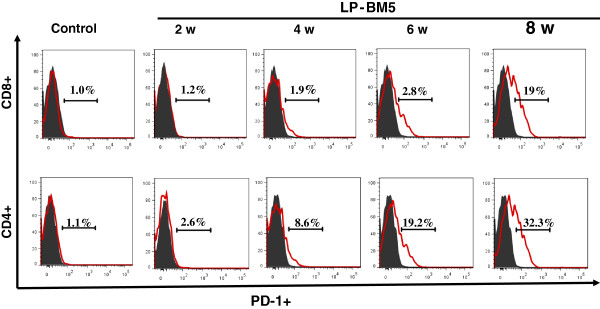
**Elevated levels of programmed death-1 on T-lymphocytes during chronic retroviral infection.** C57BL/6 mice were infected with LP-BM5 and monitored for the expression of programmed death-1 (PD-1) on circulating CD4^+^ and CD8^+^ T-cells at the indicated time points. Peripheral blood mononuclear cell preparations from individual animals were stained with CD3- allophycocyanin (APC)-Cy7, CD4-eFlour450, CD8-PE-Cy7 and PD-1- fluorescein isothiocyanate (FITC) for flow cytometric analysis. Representative histograms showing the percentage of PD-1 expression on CD4^+^ or CD8^+^ T-cells at the indicated time points are presented.

The inhibitory co-receptor PD-1 plays an important role in regulating functional exhaustion of virus-specific CD8^+^ T-cells during chronic infections [16]. Functional impairment of T-cells is characteristic of many chronic murine and human viral infections, including HIV/AIDS, due to the engagement of normal immune downregulatory mechanisms, such as the PD-1/PD-L pathway [17]. In the present study we next determined levels of PD-1 expression on circulating T-cells in susceptible C57BL/6 mice with MAIDS. Uninfected and LP-BM5-infected mice were bled at the indicated time points and PBMC were stained with mAbs. Analysis for PD-1 expression on CD4^+^ and CD8^+^ T-cells was performed using flow cytometry (Figure 2). Results generated from these studies showed that PD-1 levels on CD4^+^ T-cells gradually increased and reached peak expression (31% ± 3.3) by 8 w p.i., whereas PD-1 expression on CD8^+^ T-cells was found to be more sudden, with its highest expression also observed at 8 w p.i. (17% ± 2.5).

### Murine retrovirus-induced acquired immunodeficiency conferred susceptibility to herpes virus brain infection onto resistant C57BL/6 mice

Previous studies have shown that MAIDS leads to an immunosuppressed state characterized by gradual loss of CD4^+^ and CD8^+^ T-cell responses [11,12]. Susceptible mice develop splenomegaly, lymphadenopathy, and B-cell lymphomas. T-cell activation in MAIDS has also been reported to lead to aberrant Th2-type cytokine patterns. After initial activation of both Th1 and Th2 cell responses, the production of Th1 cytokines (IFN-γ and IL-2) falls, while Th2 cytokines (IL-4 and IL-10) rise [18]. It has been postulated that this Th1 to Th2 shift is required for progression to MAIDS. Furthermore, this retroviral infection increases susceptibility to other infections and has been used to study opportunistic parasites and fungi [19-21]. Normal C57BL/6 mice have been shown to be resistant to HSV-1 encephalitis when inoculated through the i.n. route. This resistance has been attributed to a number of factors including the herpes resistance locus (Hrl) on mouse chromosome 6 [22]. In the present study, we examined whether immunodeficiency and lymphopenia, as well as immune exhaustion due to PD-1 expression on T-cells, in MAIDS animals would render them susceptible to HSV-1 brain infection. LP-BM5-infected mice were challenged with i.n. HSV-1 infection at various stages of MAIDS disease development. C57BL/6 mice infected with either LP-BM5 or HSV-1 alone served as controls for testing the presence of HSV-1 in the brain. Brain tissue samples were harvested and divided into cortex, cerebellum, sub-cortex, and brain stem. Trigeminal ganglia (TG) were also collected. The presence of HSV-1 was determined by both real-time PCR for HSV-1 gD gene expression and virus recovery from tissue homogenates on rabbit skin fibroblast monolayers. Data shown in Table 1 suggest that MAIDS mice developed susceptibility to HSV-1 brain infection by 6 w p.i., with 83% of the mice testing positive by virus recovery assay. This susceptibility to HSV-1 brain infection persisted until 8 w p.i., a time point when 76% of the mice tested positive. Samples from other brain regions such as cortex, cerebellum and sub-cortex tested negative for presence of virus by both methods.

**Table 1:** LP-BM5 disease progression increases susceptibility of C57BL/6 mice to herpes simplex virus brain infection

**LP-BM5**	**Trigeminal ganglia**		**Brain stem**	
**(weeks post-infection)**	**(% positive)**		**(% positive)**	
	**Virus recovery**	**RT PCR**	**Virus recovery**	**RT PCR**
0 (n = 6)	100	100	0	0
2 (n = 3)	100	100	0	0
4 (n = 3)	100	100	0	0
6 (n = 6)	100	100	83	67
8 (n = 13)	100	100	76	53

Susceptibility of mice with murine acquired immunodeficiency syndrome (MAIDS) to herpes simplex virus (HSV) brain infection; LP-BM5-infected C57BL/6 mice with MAIDS were super-infected with HSV at the indicated time points. Seven days post-HSV infection, the animals were sacrificed and tissues were harvested. The tissue was then divided into cortex, sub-cortex, brain stem, and trigeminal ganglia (TG). Data presented represents virus recovery from brain stem and TG. Data are expressed as percent positive in specific tissue. Total RNA extracted from trigeminal ganglia and brain stem of three to five mice/time point was reverse-transcribed and examined for the expression of HSV gD mRNA.

### LP-BM5-induced T-cell loss and susceptibility to herpes virus brain infection were mediated through PD-1

**Figure 3:**
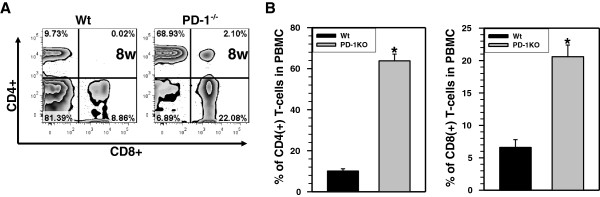
**T-cell loss during murine acquired immunodeficiency syndrome was mediated through programmed death-1.** Wild-type (Wt) and programmed death-1 (PD-1) knockout (KO) mice infected with LP-BM5 were assessed for lymphopenia by examining peripheral blood mononuclear cell (PBMC) preparations at 8 w post-infection (p.i.). PBMC were stained with CD4-eFlour450, CD8-PE-Cy7 and CD3-APC-Cy7 mAbs and analyzed by flow cytometry. **(A)** Representative flow cytometry contour plots depicting the ratio of CD4^+^ and CD8^+^ T-cells from Wt and PD-1 KO animals infected with LP-BM5 are shown. **(B)** The percentages of CD4^+^ and CD8^+^ T-cells in the PBMC preparations were determined at 8 w p.i. Data show the mean (± standard error) percentage of T-cells pooled from three independent experiments. ^*^P <0.01 versus Wt.

In the next experiments, we infected PD-1 KO C57BL/6 mice with LP-BM5 to determine if susceptibility to HSV-1 brain infection was due to immune exhaustion. As shown in Figure 3A, when compared to LP-BM5-infected, wild-type (Wt) C57BL/6 mice, PD-1 KO mice infected with LP-BM5 did not exhibit the usual loss of T-cells in peripheral blood at 8 w p.i. The PD-1 KO animals had an increased percentage of circulating CD4^+^ and CD8^+^ T-cells. In Figure 3A, a representative flow cytometric analysis of PBMC collected from PD-1 KO and Wt mice infected with LP-BM5 at 8 w p.i. is shown. Interestingly we also observed the presence of double-positive T-cells (CD4^+^CD8^+^) in PD-1 KO animals infected with LP-BM5. In HIV studies, double-positive cells such as these have been found to generate more than 55% of CD8^+^ T-cell antigen recognition and effector responses [23]. Further studies are required to identify the role of these cells in this particular disease model. The results obtained from these experiments display a significant difference in circulating CD4^+^ and CD8^+^ T-cells when compared to Wt mice (Figure 3B). This increased level of T-cells in the periphery led us to test if LP-BM5-infected PD-1 KO mice were susceptible to HSV-1 brain infection at 8 w p.i. As shown in Table 2, PD-1 KO mice retained resistant to HSV-1 brain infection implying that T-cell exhaustion, as determined by PD-1 expression, was responsible for the increased susceptibility.

**Table 2:** LP-BM5-infected programmed death-1 knockout animals retain resistance to herpes simplex virus brain infection

**LP-BM5**	**Trigeminal ganglia**		**Brain stem**	
**(8 weeks post-infection)**	**(% positive)**		**(% positive)**	
	**Virus recovery**	**RT PCR**	**Virus recovery**	**RT PCR**
C57BL/6 (n = 8)	100	100	100	100
PD-1 KO (n = 7)	100	100	00	00

LP-BM5-infected programmed death-1 (PD-1) knockout (KO) animals were resistant to herpes simplex virus (HSV) brain infection. PD-1 KO and C57BL/6 mice infected with LP-BM5 (8 w post-infection) were super-infected with HSV. At 7 d post-HSV infection animals were sacrificed and brain tissues were harvested. Data shown represent virus recovery from the brain stem and trigeminal ganglia. Data are expressed as percentage of positive tissues. Total RNA extracted from the trigeminal ganglia and brain stem of three to five mice/time point was reverse transcribed and examined for the expression of gD mRNA.

### Dual infection promoted immune cell infiltration into the brain and its associated microglial cell activation

**Figure 4:**
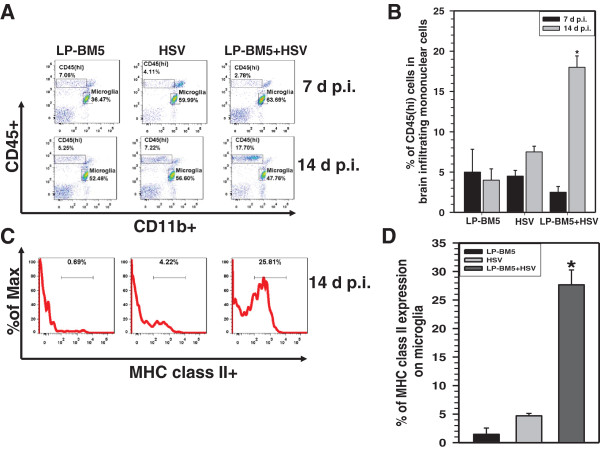
**Dual infection triggered brain infiltration by peripheral immune cells and corresponding microglial cell activation.** Single cell suspensions of brain tissue obtained from LP-BM5-, herpes simplex virus (HSV)- and LP-BM5 + HSV-infected mice (three to five animals per time point) were banded on a 70% Percoll cushion. Brain leukocytes were collected and labeled with PE-Cy5-conjugated Abs specific for CD45, AF700-labeled anti-CD11b, and allophycocyanin (APC)-labeled major histocompatability complex (MHC) class II. Analysis was done by using flow cytometry (FacsCanto, BD Biosciences, CA), with FlowJo software (TreeStar, Inc.). **(A)** The dot plots presented are representative of three experiments at 7 and 14 d post-infection (p.i.). The CD45^hi^ population, representing peripheral immune cells, are clearly differentiated from the CD45^int^ resident microglial cells. **(B)** The percentages of CD45^hi^ cells among each group pooled from three independent flow cytometry experiments at 7 and 14 d p.i. with HSV are shown. **(C)** CD45^int^CD11b^+^ microglia were analyzed for upregulation of the activation marker MHC class II. Representative histograms from three independent experiments are shown. **(D)** The percentages of MHC class II expression on microglial cells at 14 d p.i. are shown. ^*^P <0.01 versus HSV infection alone.

In previous studies, using a BALB/c mouse model of HSV-1 brain infection, we showed that peripheral immune cells infiltrate the brain and that they persist for a prolonged period of time [24]. In another model of herpes virus brain infection, our studies showed that brain-resident microglial cells were activated in response to IFN-γ secreted from infiltrating T-cells [25]. In the current study, we next assessed the presence of peripheral immune cells within brains of MAIDS mice super-infected with HSV-1. Brain-infiltrating leukocytes from HSV-1-, LP-BM5-, and dual-infected groups were isolated on a Percoll gradient at 7 and 14 d p.i. The leukocytes were subsequently stained with flourochrome-tagged mAbs, CD45, CD11b and MHC class II. Flow cytometric analysis of CD45- versus CD11b-expressing cells differentiates CD45^hi^ peripheral immune cells from CD45^int^ resident microglial cells, as shown in Figure 4A. At 7 d p.i. with HSV-1, minimal peripheral immune cell infiltration of the brain of MAIDS mice as indicated by CD45^hi^ cells was observed (Figure 4A, upper panel). However, the percentage of CD45^hi^ cells increased to approximately 18% ± 1.14 in dual-infected animals by 14 d p.i. (Figure 4A, lower panel). The frequency of CD45^hi^ cells was significantly higher in MAIDS mice at 14 d p.i. with HSV-1 when compared to either of the control groups that were infected singly, suggesting super-infection with HSV-1 drove peripheral immune cell infiltration. We also assessed the activation of resident microglia by determining levels of expression of MHC class II, an activation marker, by specifically gating on the CD45^int^CD11b^+^ cell population. Results obtained from these studies demonstrate that dual infection induced expression of MHC class II on microglial cells (Figure 4C), MHC class II expression on microglia in a quiescent brain is <5% and its expression is considered a surrogate marker for microglial activation [25]. Expression levels were significantly higher on microglia isolated from dual-infected mice when compared to either control group (Figure 4D).

### T-cell reconstitution was lethal to MAIDS mice with opportunistic brain infection

**Figure 5:**
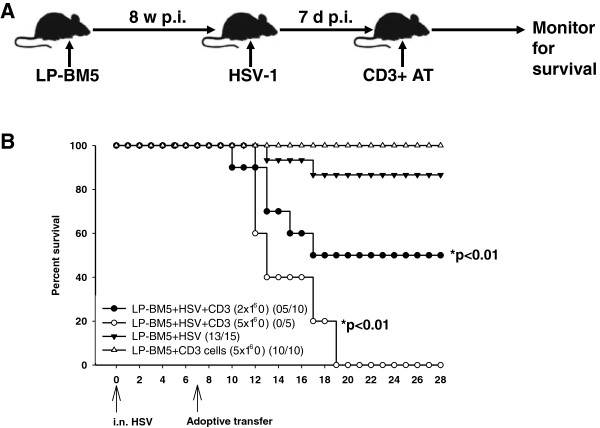
**T-cell reconstitution was lethal to herpes simplex virus-infected mice with murine acquired immunodeficiency syndrome. (A)** Schematic representation of the experimental design. In brief, LP-BM5-infected murine acquired immunodeficiency syndrome (MAIDS) mice at 8 w post-infection were infected i.n. with 1 × 10^5^ PFU of herpes simplex virus (HSV). At 7 d post-HSV infection, 2 × 10^6^ or 5 × 10^6^ CD3^+^ T-cells isolated from HSV-primed, major histocompatability complex (MHC)-matched C57BL/6 mice were intravenously transferred into the dual-infected MAIDS animals. **(B)** Following immune reconstitution the recipients were monitored for survival. Survival data are expressed as percent of mice in each group surviving at the indicated time point, followed over the time course of the experiment. ^*^P <0.01, log rank test.

Prolonged T-cell deficiency typically results in increased susceptibility to microbial infection, and normalization of T-cell numbers usually benefits the host through restoration of protective immunity. In the above studies, we demonstrated that susceptibility of MAIDS mice to HSV-1 brain infection was dependent upon immune exhaustion and lymphopenia. To recapitulate lymphopenic AIDS patients undergoing immune reconstitution, and to study its consequences within brains harboring OI, we transferred different doses (2 × 10^6^ and 5 × 10^6^ cells) of purified CD3^+^ T-cells from HSV-1-primed donors into MAIDS mice that had previously been infected with HSV-1 7 d earlier (Figure 5A). Interestingly, MAIDS mice harboring herpes virus brain infection (>85%) survived through the study period, whereas T-cell dose-dependent mortality was observed among the adoptive transfer recipients. All mice receiving immune reconstitution with the higher dose succumbed (n = 5, *P* <0.00982, log rank test), while we observed >50% mortality in mice receiving the lower dose of T-cells (n = 10, *P <*0.00982, log rank test) (Figure 5B). During previous studies, we performed adoptive transfer of immune cells from HSV-1-primed donors into infected mice at 4 d p.i. and determined that these donor cells were not an additional source of virus [24].

### Lethal disease was associated with exacerbated neuroinflammation following immune reconstitution

**Figure 6:**
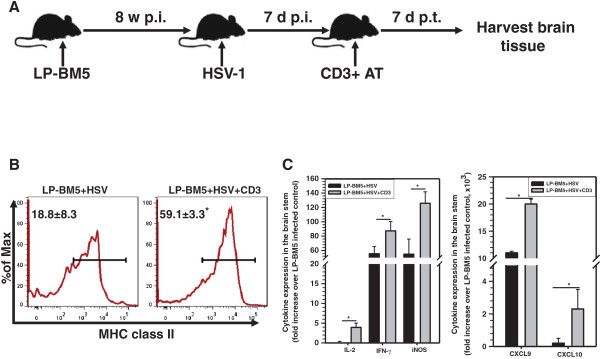
**Lethal disease was associated with exacerbated neuroinflammation. (A)** Schematic representation of the experimental design. In brief, C57BL/6 mice with murine acquired immunodeficiency syndrome (MAIDS) (8 wks post-LP-BM5 infection) were super-infected with herpes simplex virus (HSV)-1 through i.n. inoculation. T-cell reconstitution was induced 7 d post-HSV infection through adoptive transfer of 5 x 10^6^ CD3^+^ cells obtained from primed donor animals via tail vein injection. Brain tissues were harvested from the LP-BM5 + HSV and LP-BM5 + HSV + CD3AT groups 7 d post-adoptive transfer (p.t.). **(B)** Brain leukocytes were collected and labeled with PE-Cy5-conjugated antibodies specific for CD45, AF700-labeled anti-CD11b, and allophycocyanin-labeled major histocompatability complex (MHC) class II. Brain-resident CD45^int^CD11b^+^ microglial cells were analyzed for the activation marker MHC class II. Histograms showing pooled data from three independent experiments are shown. Data are presented as mean ± standard error percentage of CD45^int^CD11b^+^ cells displaying MHC class II expression. (**C**) mRNA expression for the indicated proinflammatory mediators was assessed using real-time quantitative reverse-transcribed PCR on total RNA extracted from brain stem homogenates at 7 d p.t. Levels of mRNA were normalized to hypoxanthine guanine phosphoribosyl transferase-1 and are presented as mean ± SD normalized to LP-BM5 control from pooled data obtained using five animals per group from three independent experiments. ^*^P <0.05 versus LP-BM5 + HSV-infected mice.

Disease mechanisms responsible for CNS-IRIS have not been elucidated, in part due to the strict host requirements for HIV-1. It is becoming apparent that IRIS and CNS-IRIS are highly diverse syndromes with multiple possible disease mechanisms and processes. A recent study demonstrated a correlation between neuronal dysfunction and increased production of chemokines responsible for lymphocyte recruitment, as well as chemokines produced by activated lymphocytes [26]. To examine the contribution of immune reconstitution to neuroinflammation in MAIDS mice harboring HSV-1 brain infection, we repeated the above experiment and harvested brain tissues at 7 d post-immune reconstitution, prior to death. This experimental outline is depicted in Figure 6A. Previously, we reported that infiltrating T-cells mediate exacerbated neuroimmune responses. To address the effect of immune reconstitution on resident microglial cells, distinctly identified as CD45^int^CD11b^+^ cells, we analyzed these cells for activation by detecting MHC class II expression. A significant increase in the levels of expression of MHC class II on microglia recovered from T-cell recipients was observed when compared to animals not receiving adoptive transfer (Figure 6B). mRNA expression levels of the proinflammatory mediators IL-2, IFN-γ, iNOS, and IFN-γ-inducible chemokines, CXCL9 and CXCL10, were determined using real-time qPCR. A corresponding significant increase in Th1 cytokines and chemokine expression was observed among MAIDS mice that received adoptive transfer following HSV-1 infection (Figure 6C).

### CNS-IRD was independent of PD-1 expression on T-cells

**Figure 7:**
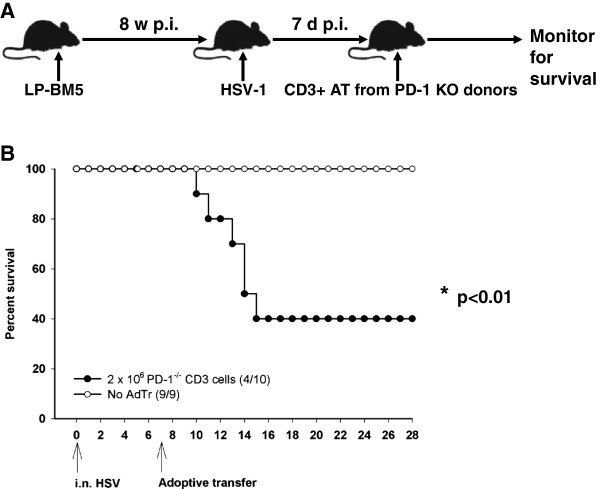
**Reconstitution of T-cells from programmed death-1 knockout donors was immunopathogenic. (A)** In brief, C57BL/6 mice were infected with the LP-BM5 retrovirus mixture for 8 weeks to induce murine acquired immunodeficiency syndrome and brain super-infection with herpes simplex virus (HSV)-1 was established through i.n. inoculation. Immune reconstitution was induced following adoptive transfer of 2 × 10^6^ CD3^+^ cells obtained from primed programmed death-1 knockout donor animals. **(B)** Following immune reconstituion the recipients were monitored for survival. Survival data are expressed as percent of mice in each group at the indicated time point. *P <0.01, log rank test.

AIDS patients who develop IRIS have been shown to express high levels of PD-1 on their T-cells [27]. PD-1 expression on CD4^+^ or effector memory T-cells could mean hyperactivation of a functionally impaired peripheral immune system that may quickly reverse following initiation of HAART [28]. Several studies have shown that PD-1 expression on T-cells is an indicator of immune activation. Both monkey and mouse model studies have revealed that blocking the PD-1/PD-L1 pathway has beneficial effects, preventing peripheral immune activation and facilitating more effective virus control [27,29,30]. In order to assess the role of PD-1 in neuroimmunopathology in our model, we performed adoptive transfer experiments similar to what was described above, albeit using primed T-cells from PD-1 KO donors (Figure 7A). In these experiments, we still observed 60% mortality in mice receiving a 2 × 10^6^ dose of T-cells (n = 10, *P <*0.00982, log rank test) from PD-1 KO animals (Figure 7B). A finding, which implies that unlike opportunistic brain infection, reconstitution disease seen in MAIDS mice harboring opportunistic infection was independent of PD-1 expression on T-cells.

## Discussion

CNS-IRIS is a major emerging health concern due to worldwide accessibility of HAART, along with the prolonged lifespan of HIV-infected individuals. However, the immunopathogenic mechanisms responsible are still poorly understood. Although human studies would be most relevant to address this disease, they are severely limited by numerous factors including availability of tissue samples and variability among patient cohorts. Unfortunately, our understanding of the molecular and cellular interactions driving destructive neuroimmune responses during CNS-IRIS is hampered due to lack of an appropriate animal model. Data presented here provide evidence that OI of MAIDS mice combined with T-cell immune reconstitution provides an accessible animal model for investigating neuropathologic immune reconstitution.

IRIS is most commonly seen in patients with severe CD4^+^ T cell lymphopenia (<100 cells/mL) at the initiation of treatment, often in the presence of an identifiable OI. In this study, we examined experimental immune reconstitution disease of the CNS using T-cell repopulation of lymphopenic murine hosts harboring opportunistic HSV-1 brain infection and assessed its potential to model CNS-IRIS. Murine models have been used extensively to study the pathogenesis of HSV-1 encephalitis and it is well-established that the genetic background of mice dictates disease outcomes. For example, HSV-1 17^+^ strain produces encephalitis in BALB/c mice when inoculated through the i.n. route, but the same virus does not produce brain disease in C57BL/6 mice when administered through an identical i.n. route. Resistance of C57BL/6 mice to HSV-1 encephalitis has been attributed to a number of factors including the Hrl on mouse chromosome 6 [22]. Data presented here demonstrated that C57BL/6 MAIDS mice became susceptible to HSV-1 brain infection due to LP-BM5-induced immunodeficiency and an exhausted T-cell phenotype. Previous studies have shown that a lack of coordinated responses from CD8^+^ T-cells and natural killer cells facilitated HSV-1 spread into brains of C57BL/6 mice [31]. MAIDS models have been extensively used to understand the pathogenesis of opportunistic infections [18,20,21,32]. In our model of LP-BM5-induced immunodeficiency, otherwise resistant C57BL/6 mice became sensitive to opportunistic brain infection.

Although LP-BM5 infection also induces lymphoproliferation within secondary lymphoid organs, we detected a sharp decline in circulating CD4^+^ and CD8^+^ T-cells. This finding is similar to a previous result that utilized a Thy1.2 marker to demonstrate loss of T-cells in the blood [33]. Interestingly, the remaining circulating T-cells were found to express high levels of PD-1, suggesting functional exhaustion. It has been demonstrated that inhibitory receptors, such as PD-1, are expressed at elevated levels on both CD4^+^ and CD8^+^ T-cells in subjects with chronic HIV-1 infection, and diminished function of these cells may contribute to ineffective control of HIV-1 replication [34,35]. Furthermore, in a simian immunodeficiency (SIV) model, blockade of the PD-1 pathway *in vivo* increased SIV-specific T-cell function, decreased SIV viral loads (VLs), decreased opportunistic infections, and increased the life span of infected macaques [36]. Previous studies using the LP-BM5 model have shown that blocking the PD-1 pathway substantially altered MAIDS progression [37]. In this study, we demonstrated that following inhibition of the PD-1/PD-L1 pathway through the use of PD-1 KO animals, lymphopenia associated with LP-BM5 infection was not observed and these KO mice retained their normal resistance to HSV-1 brain infection.

Characteristics of particular inciting pathogens may also affect immunopathology in IRIS. IRIS is most often associated with CD4^+^ Th1-mediated immune responses; however, both CD4^+^ and CD8^+^ effector T-cells are involved. In this study, we describe a new model of reconstitution disease that recapitulates the fundamental immunologic scenario of opportunistic infection-associated CNS-IRIS: T-cell reconstitution of lymphopenic hosts harboring active viral brain infection. Our data suggest that susceptibility to IRD may be a common property of microbial-infected immunodeficient hosts undergoing T-cell reconstitution. Indeed, induction of inflammatory disease has also been described following adoptive transfer of CD4^+^ T-cells into *Pneumocystis carinii*–exposed *SCID* mice [38] and Mycobacterium-infected lymphopenic mice [39]. We used T-cells from HSV-1-primed mice in adoptive transfer experiments based on our previous findings that only activated T-cells infiltrate brains [24].

An essential feature of CNS-IRIS is infiltration of the brain with activated T-cells, which occurs in an attempt to control underlying CNS infection. While T-cell transfer into HSV-1–infected MAIDS mice triggers disease, no IRIS-like symptoms were seen in MAIDS mice that harbored herpesvirus brain infection without adoptive transfer despite having some cellular infiltration. These studies show that pathogen-driven neuroimmune responses are important in development of what is termed as “unmasking” CNS-IRIS. Although a wide variety of opportunistic pathogens have been associated with CNS-IRIS, the neuroimmunoregulatory networks involved remain to be elucidated using animal models because they are difficult to address through clinical studies.

Using a Mycobacterial model of IRIS in TCRα^−/−^ mice, it was demonstrated that Ag-specific Th1 responses are deleterious. Rapid induction of serum nitric oxide was observed after transfer of Wt CD4 T-cells (but not IFNγ^−/−^ CD4 T cells) into infected TCRα^−/−^ recipients implying that IFN-γ-inducible downstream cytokines were produced in response to T-cell reconstitution. Similarly, in this study, we observed significantly elevated levels of Th1 proinflammatory mediators within brains of mice receiving T-cells. Indeed, previous studies from our laboratory investigating chronic neuroimmune responses following herpesvirus infections have revealed that brain-resident microglia respond to infiltrating T-cell produced IFN-γ [24,25]. It has been documented in well-studied cohorts of cryptococcal CNS-IRIS that, at the site of inflammation in the CSF, numerous cytokines are increased, including IFN-γ (2.5-fold increase), TNF-α (threefold increase), and IL-6 (twofold increase) [40]. Here, we also provided evidence of exacerbated microglial activation following T-cell reconstitution along with increased expression of Th1 cytokines in the brains of mice that received T-cells.

Hyperimmune activation, as determined by PD-1 expression on T-cells, has been shown to be a strong predictor of disease progression, and the reduction of hyperimmune activation following anti-PD-1 antibody treatment could contribute to enhanced survival. This phenomenon has been demonstrated in multiple studies in which blocking of the PD-1/PD-L1 pathway was shown to be advantageous, using both SIV [29] and humanized mouse [30] models. In the present study, MAIDS mice harboring HSV-brain infection were equally susceptible to immune reconstitution using T-cells obtained from PD-1 KO donors.

In recent years, the MAIDS system has fallen out of favor as a model of HIV-1 infection because B-cells were found to be infected with two of the three viruses in the LP-BM5 mixture, and a B-cell neoplastic disorder develops in the later stages of disease. The caveats of using this model are an inevitably fatal syndrome characterized by splenomegaly, lymphadenopathy, and a progressive loss of B- and T-cell responses to antigens and mitogens. Infected animals become sluggish with enlarged lymph nodes probably creating obstruction and discomfort. Although insights into the pathogenesis of CNS-IRIS emerging from this study may have important implications for the understanding of viral etiology-associated CNS-IRIS, the model itself does not fully explain all of the diverse manifestations of this disease.

## Conclusion

Our results reveal that T-cell reconstitution of severely lymphopenic hosts harboring opportunistic viral brain infection can lead to adverse outcomes. Additionally, dual infection alone was not found to be lethal, but T-cell reconstitution produced exaggerated disease, neuroimmune responses, microglial activation, and increased expression of proinflammatory cytokines within the CNS. Taken together, this model of opportunistic viral brain infection is useful to investigate immune reconstitution disease of the CNS.

## Abbreviations

Ab: Antibody; Bp: Base pairs; APC: Allophycocyanin; AT: Adoptive transfer; CNS: Central nervous system; CPE: Cytopathic effect; Ct: Cycle threshold; CXCL: Chemokine (C-X-C motif) ligand; DMEM: Dulbecco’s modified Eagle’s medium; FBS: Fetal bovine serum; HAART: Highly active antiretroviral therapy; HBSS: Hank’s balanced salt solution; HEPES: 4-(2-hydroxyethyl)-1-piperazineethanesulfonic acid; HPRT-1: Hypoxanthine guanine phosphoribosyl transferase-1; Hrl: Herpes resistance locus; HSV: Herpes simplex virus; IFN: Interferon; IL: Interleukin; i.n.: Intranasal; iNOS: Inducible nitric oxide synthase; i.p.: Intraperitoneal; IRD: Immune reconstitution or restoration disease; IRIS: Immune reconstitution inflammatory syndrome; MAIDS: Murine acquired immunodeficiency syndrome; IRS: Immune reconstitution syndrome; KO: Knockout; mAb: Monoclonal antibody; MAIDS: Murine acquired immunodeficiency syndrome; MHC: Major histocompatability complex; MuLV: Murine leukemia virus; OI: Opportunistic infections; PBMC: Peripheral blood mononuclear cells; PD-1: Programmed death-1; p.i.: Post-infection; RBC: Red blood cells; qPCR: Quantitative polymerase chain reaction; RPMI: Roswell Park Memorial Institute; SIV: Simian immunodeficiency; TG: Trigeminal ganglia; Th: T-helper; Wt: Wild-type.

## Competing interests

The authors declare that they have no competing interests.

## Authors’ contributions

MBM and JRL conceived and designed the experiments. MBM, SJS and SH performed the experiments. MBM, SJS and JRL analyzed the data. MBM and JRL wrote the paper. All authors read and approved the final manuscript.
